# City lockdown and nationwide intensive community screening are effective in controlling the COVID-19 epidemic: Analysis based on a modified SIR model

**DOI:** 10.1371/journal.pone.0238411

**Published:** 2020-08-28

**Authors:** Tailai Peng, Xinhao Liu, Hefeng Ni, Zhe Cui, Lei Du

**Affiliations:** 1 Department of Anesthesiology and Translational Neuroscience Center, West China Hospital, Sichuan University, Chengdu, Sichuan, China; 2 University of Chinese Academy of Sciences, Beijing, China; Faculty of Science, Ain Shams University (ASU), EGYPT

## Abstract

**Background:**

In December 2019, an outbreak of COVID-19 epidemic occurred in Wuhan, China and infection spread rapidly around the world. To limit the rapid spread locally and nationwide, the Chinese government locked down Wuhan city on January 23 and began implementing nationwide intensive community screening on February 16.

**Method:**

To assess the effectiveness of city lockdown and intensive community screening, we built a modified SIR model by introducing an α value into the classic SIR model. The α value represents the proportion of infected individuals who are not effectively isolated from susceptible individuals at a given time point.

**Results:**

The accuracy of the modified SIR model was validated using data from Guangdong and Zhejiang provinces. The lockdown of Wuhan city substantially reduced the α value for the rest of China excluding Hubei province, while only slightly reducing the α value for the city itself. Intensive community screening rapidly reduced the α value for Wuhan.

**Conclusion:**

City lockdown was efficient in controlling the spread of the epidemic from Wuhan to the rest of the country. Nationwide intensive community screening was extremely effective in suppressing the spread locally within Wuhan city. These experiences should urgently be shared with other countries to help halt the progressing worldwide pandemic.

## Background

An outbreak of novel coronavirus (SARS-CoV-2) infection occurred in Wuhan city, China in December 2019 [1]. The World Health Organization (WHO) has named the disease caused by SARS-CoV-2 infection as coronavirus disease 2019 (COVID-19). No specific medication for SARS-CoV-2 infection is currently available, only supportive care [2]. On July 16, 2020, the cumulative number of people with COVID-19 exceeded 13,150,000 around the world, and the official reported death toll exceeded 574,000 [3]. Although cross-species transmission of SARS-CoV-2 has not been clarified, rapid person-to-person transmission has been linked to the Huanan Wholesale Seafood Market [4]. The outbreak began immediately before the traditional Chinese New Year, when mass migration occurs every year, facilitating the spread of the epidemic. Making the situation worse, Wuhan is one of the largest cities in China, with a population of 11 million and an extensive transportation system including flights, express trains and local buses. The epidemic overwhelmed the healthcare system of Wuhan city in early February and caused more than 3000 deaths of healthcare workers in China by early March [5]. To halt this serious public health event, the Chinese government implemented two stringent mitigation measures: Wuhan city lockdown at the end of January and nationwide intensive community screening in early February.

The present study was carried out in an early stage of the epidemic in China and was meant to measure the effectiveness of these two measures through a modified SIR model and to make predictions about the first wave of the epidemic in China. First, we validated the accuracy of the model using real-world data from Guangdong and Zhejiang provinces in China. Second, we used the validated model to evaluate the effectiveness of the two measures. Our model predicted that the first wave of the epidemic would come under control in early June, which closely approximates real-world data.

## Methods

### Data source

The reported cases of COVID-19 were collected from December 8, 2019 to March 12, 2020 using Tencent real-time tracking [6], Ifeng real-time tracking [7] and data from the National Health Commission of the People’s Republic of China [8]. Our data include new infections, new cures, new deaths, daily recoveries, cumulative diagnoses, cumulative discharges, and cumulative deaths each day. Because our data were on a daily basis, our model was iterated to produce daily results.

### Establishing a classical SIR model

The present study used the classic SIR model to build a mathematical model, where S represents susceptible individuals; I, infected individuals; and R, recovered individuals.

Suppose *i* (*t*), *s* (*t*) and *r* (*t*) represent the numbers of infected, uninfected and recovered individuals, respectively, at time *t*. Meanwhile, the effective daily infection rate *β* is defined as *λ*×*p*, where *λ* is the number of infected and healthy individuals’ effective contacts with infected individuals per day, and *p* is the infection probability during each contact. The model equations were established to be

$$i(t+\Delta t)-i(t)=i(t)\times \beta \times \frac{s(t)}{N}\times \Delta t-i(t)\times \mu \times \Delta t$$ (1)

$$s(t+\Delta t)-s(t)=-i(t)\times \beta \times \frac{s(t)}{N}\times \Delta t$$ (2)

r(t+Δt)−r(t)=i(t)×μ×Δt (3)

where $\frac{s(t)}{N}$ represents the proportion of healthy individuals involved in the effective contacts, since only they can transmit the disease.

### Calculation of infection probability *p*

We calculated *p* using the first available local data from Wuhan city (January 18, 2020) and extending until the city lockdown on January 23, 2020. Infection number was calculated as the cumulative number of confirmed cases minus the cumulative number of hospital discharges and deaths. Based on the initial *i* (*t*) and real-time *i* (*t*) during this period, the value of *p* in *i*(*t*) was found.

Since the initial *N*≈*s*(*t*), we can simplify (1) to get:

$$\frac{di}{dt}=(\beta -\mu )\times i(t).$$ (4)

According to (4), the expression *i*(*t*) can be obtained:

$$i(t)=C\times e^{(\beta -\mu )t}.$$ (5)

Because *i*(0) = 1, then *C* = 1 and

$$i(t)=e^{(\beta -\mu )t}.$$ (6)

Substituting *β* = *λ*×*p* into (6) gives

$$i(t)=e^{(\lambda p-\mu )t}.$$ (7)

We assumed the number of effective daily contacts per infected person was 5 (i.e. *λ* = 5), according to the estimation by Kucharski [9]. According to Chinese statistics, the average length of hospitalization was 20 days in Wuhan city and 9 days outside Hubei, so the average length of hospitalization was $\lfloor \frac{20+9}{2}\rfloor =14$ days. *μ* (*Mu*) is defined as recovery rate and thus, *μ* (*Mu*) = 1/14. Because the first case was found on December 8, 2019, the value of “*t”* was the difference between that date and the given date.

These considerations led to the fitted objective function

$$min\sum_{t\epsilon T}{\left(e^{(\beta -\mu )t}-I(t)\right)}^{2}$$

where *I* (*t*) is the number of patients on t days after December 8.

Based on data from Wuhan from December 18, 2019 to December 22, 2019, we found *β* = 0.20. Since *β* = *λ*×*p*, we found *p* = 0.040 and R_0_ = 2.805. This value of *p* was determined from real-world Hubei data, so we did not calculate a 95% confidence interval for it.

### Establishing a modified SIR model

Two conditions were imposed in order to allow the above SIR model to take into account the Chinese government’s drastic measures to contain virus spread. First, isolated infected individuals were assumed to be unable to transmit the virus to others but able to recover. Second, a certain proportion of infected individuals was assumed to be able to transmit the virus to others without limitation. These two conditions would alter the daily number of effective contacts between an infected individual and healthy individuals (*λ*).

In order to capture these changes in the model, we introduced the coefficient of proportionality *α* value into the abovementioned classical SIR model. *i*(*t*)×*α* represents the patients who are not quarantined. If disease detection is effective, then *α* ≤ 1, or if there are some undetected cases, then *α* > 1 and *i*(*t*)×*α* represents both detected and undetected patients who are not quarantined. In either case, the implementation of effective measures will lead to a decrease in α, and the larger the decrease in α, the higher the proportion of patients in quarantine, suggesting better efficacy of measures.

After *α* was introduced into formulas (1)-(3), *i*(*t*)×(1−*α*)×*μ*×Δ*t* was replaced by *daily_recover_num*(*t*), which is the true number of recovered patients obtained from public databases. This led to the following formulas:

$$i(t+\Delta t)-i(t)=i(t)\times \alpha \times \lambda \times p\times \frac{s(t)}{s(t)+i(t)\times \alpha }\times \Delta t-daily\_recover\_num(t)$$ (8)

$$s(t+\Delta t)-s(t)=-i(t)\times \alpha \times \lambda \times p\times \frac{s(t)}{s(t)+i(t)\times \alpha }\times \Delta t$$ (9)

r(t+Δt)−r(t)=daily_recover_num(t) (10)

We used (9) to calculate the value of α, where λ = 5, other parameters are defined by real-world data, and t and t+Δt refer to two consecutive days. We defined the target date as the time point t+Δt, and the *α* value of t+Δt was defined as the average *α* value of *t* and *t*+Δ*t*. For example, to measure the *α* trend, we first calculated the true *α* value of the target day as *α*_*t*_, then we calculated the *α* value of the day before the target day as *α*_*b*_. Finally we took *α* = (*α*_*b*_+*α*_*t*_)/2 as the target day’s *α*. We reasoned that this approach might increase fit accuracy

We also predicted future daily recovery numbers (*daily_recover_num*(*t*+Δ*t*)) based on current daily recovery numbers. The resulting (*daily_recover_num*(*t*+Δ*t*) was used together with the *α* value to predict i(t). We calculated future numbers of cured people every day (*daily_recover_num*(*t*)) from the cumulative number of cured people *φ*.

### Prediction of new α values

The prediction of cumulative cases requires an α value for each day. To predict the *α* value for M days starting from day T, we selected the real *α* values for n days from day T-n until day T-1 and fitted its trend. Usually an n between 10 and 15 was required, and the trend of *α* value for the n days was used to fit the function. In the same way, we fitted and predicted the *φ* value. We used a polynomial function to fit the trends in α and *φ* values based on the “curve_fit” algorithm and its default loss function in packages of scipy.optimize and poly of numpy in Python.

### Predicting infected cases using the predicted α value and predicted daily-recovered value

Based on the *α* and *φ* values predicted above, we built an iterative model according to Eqs (8)–(10). We substituted the previous day’s *i*(*t*), *s*(*t*), and *r*(*t*) into the model, used the predicted *φ* value to calculate *daily_recover_num*(*t*), and incorporated the predicted α values and *daily_recover_num*(t). Each time we incorporated a predicted value, we obtained the next day’s *i*(*t*), *s*(*t*), and *r*(*t*). Repetition for *n* values of *α* and *daily_recover_num*(*t*) led to a prediction of the infection cases (*i*(*t*)) for the next *n* days.

## Results

### Validation of the modified SIR model

**Fig 1:**
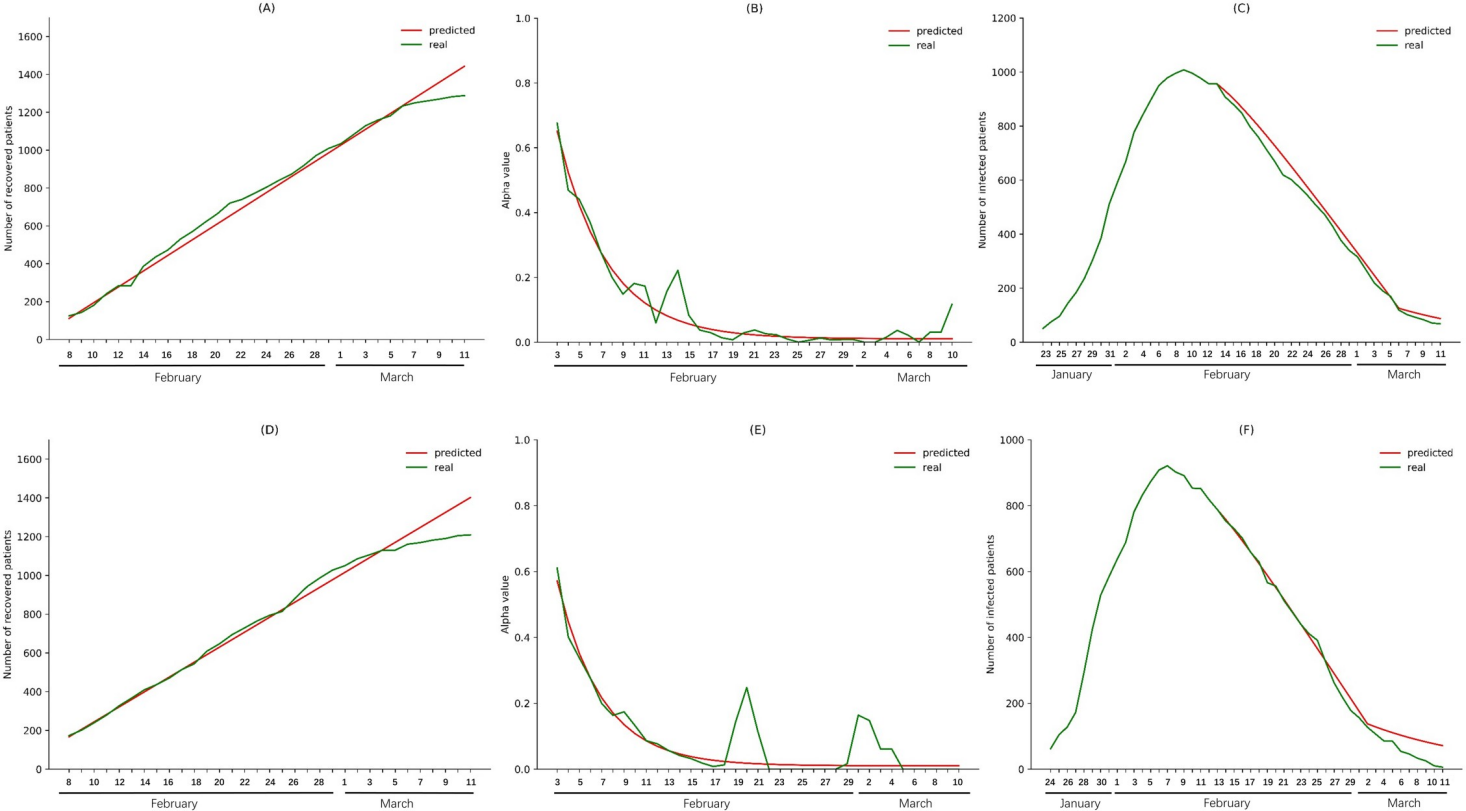
Validation of the modified SIR model using data from Guangdong and Zhejiang provinces. The *φ* values from February 3 to February 12 (before nationwide intensive community screening but after Wuhan city lockdown) were used to predict *φ* values from February 13 to March 11 for Guangdong (A) and Zhejiang (D). The *α* values from February 8 to February 12 (before nationwide intensive community screening but after Wuhan city lockdown) were used to predict *α* values from February 13 to March 10 for Guangdong (B) and Zhejiang (E). Predicted *α* values and *φ* values were used to predict the number of infections in from February 14 to March 11 for Guangdong (C) and Zhejiang (F).

We made predictions for Guangdong and Zhejiang provinces to verify the accuracy of the model. Data of Guangdong and Zhejiang from February 3 to February 12 were used to fit the trend of *φ* values, and data of Guangdong and Zhejiang from February 8 to February 12 were used to fit the trend of *α* values. Then the fitted trend was used to predict the *α* value from February 13 to March 10 and *φ* values from February 13 to March 11. The predicted *φ* values were used to calculate the values of *daily*_*recover*_*num*. Then the predicted *α* values and values of *daily*_*recover*_*num* were integrated into the modified SIR model along with the *i*(*t*),*s*(*t*), and *r*(*t*) of February 13 to predict the *i*(*t*),*s*(*t*), and *r*(*t*) on each day. As shown in Fig 1, the predicted *α* values, *φ* values and numbers of infection cases in Guangdong and Zhejiang provinces agreed well with the real numbers before March 11, and the predicted *α* values decreased sharply after the implementation of Wuhan city lockdown. These results indicate the accuracy of the model. However, the predicted cases were higher than the real infection cases in Guangdong (87 vs. 68) and Zhejiang (71 vs. 6) on March 11, which may be due to the delayed effect of nationwide intensive community screening, which began on February 16 and caused a reduction in infections starting about 10 days later (from February 27), further reducing the α value.

### Evaluation of the effectiveness of city lockdown and intensive community screening

**Fig 2:**
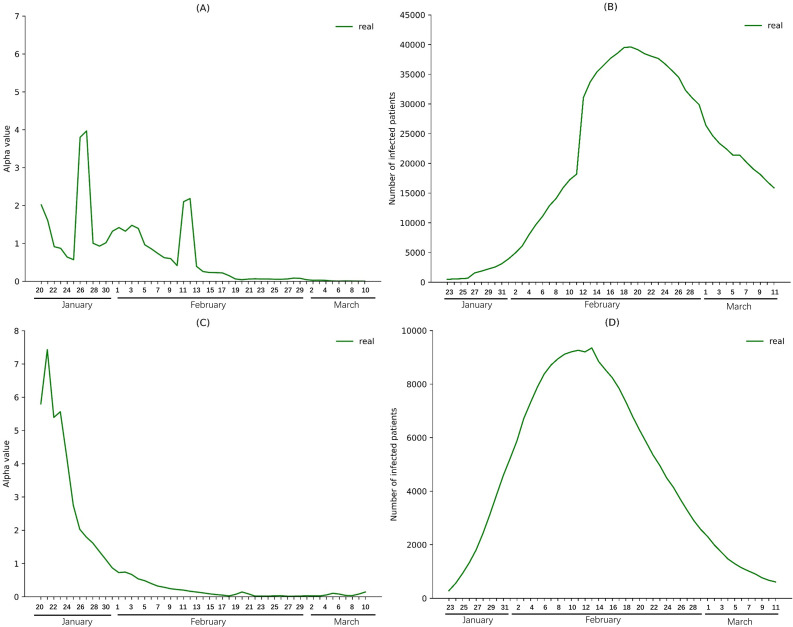
Real-world *α* values and numbers of infections in Wuhan city from January 20 to March 10 and in China excluding Hubei from January 23 to March 11. Real *α* values (A) and numbers of infections (B) in Wuhan city; real *α* values (C) and numbers of infections (D) in China excluding Hubei.

We calculated *α* values for Wuhan city and China excluding Hubei (Fig 2), and found that the *α* value of Wuhan decreased slightly after city lockdown (from 0.869 on January 23 to 0.228 on February 16). However, the *α* value of China excluding Hubei decreased steadily after January 23 (from 5.563 on January 23 to 0.064 on February 16). These results suggest that the rapid spread of the virus from Wuhan to other cities was effectively suppressed, but not the local spread in Wuhan.

The nationwide intensive community screening (starting on February 16) was associated with a significant decrease in the α value of Wuhan city (from 0.228 on February 16 to 0.003 on March 10) and a stable α value of China excluding Hubei (from 0.064 on February 16 to 0.079 on March 10). This suggests that intensive community screening significantly enhanced the effectiveness of Wuhan city isolation and kept infection levels stable in other regions. As a result, the infected cases decreased significantly in Wuhan and China excluding Hubei, from 36385 and 8163 on February 16, respectively, to 13462 and 493 on March 11 (Fig 2).

**Fig 3:**
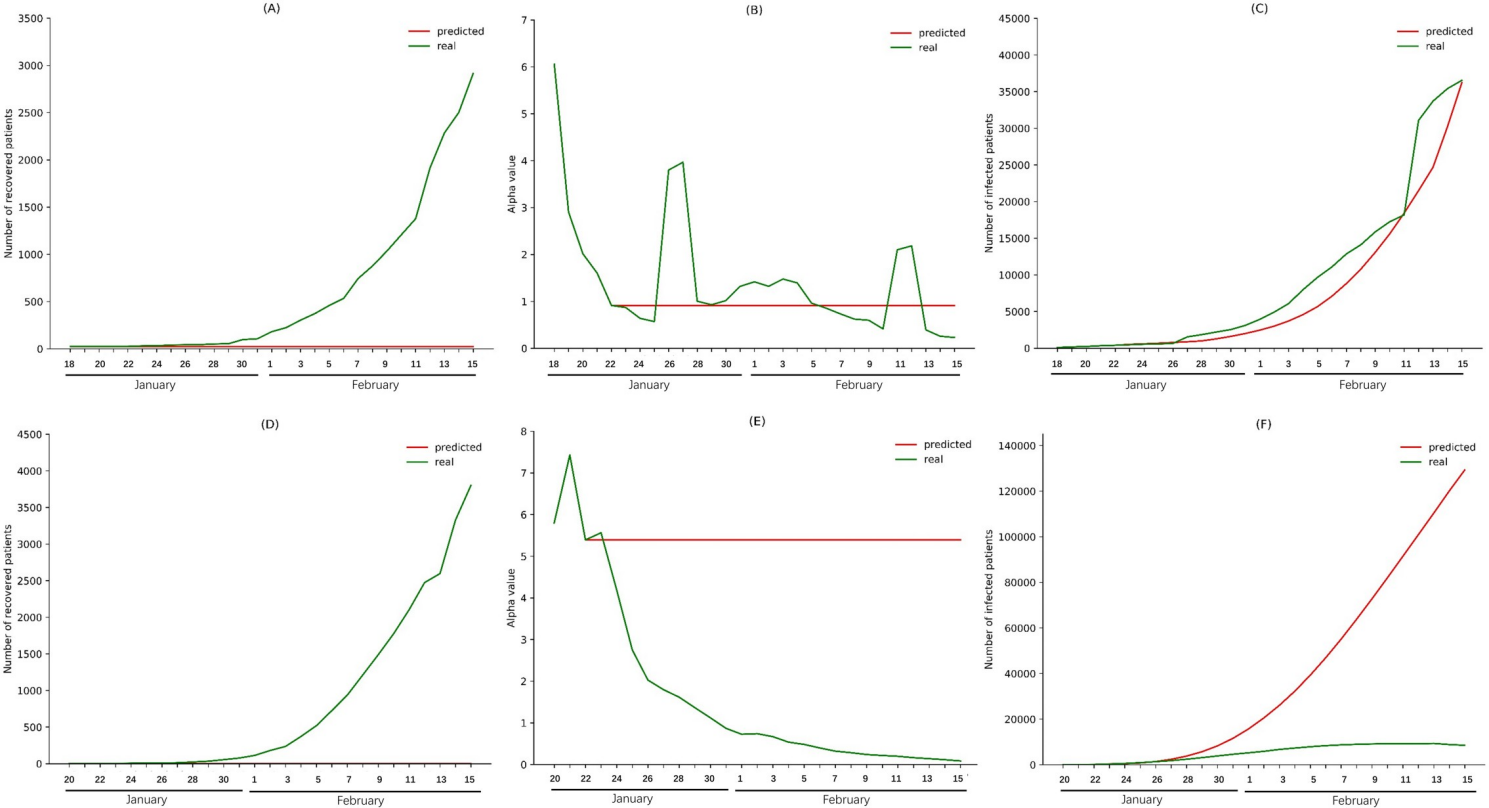
Impact of city lockdown on Wuhan city and China excluding Hubei province. (A) The *φ* values for Wuhan city at January 22 (before city lockdown) were used to predict the *φ* values from January 23 to February 15 without city lockdown. (B) The *α* values for Wuhan city at January 22 (before city lockdown) were used to predict the *α* values from January 23 to February 14 without city lockdown. (C) Predicted *α* values and *φ* values were used to calculate the number of infections in Wuhan city from January 24 to February 15. (D) The *φ* values for China excluding Hubei province on January 22 were used to predict the *φ* values from January 23 to February 15 without city lockdown. (E) The *α* values for China excluding Hubei province on January 22 were used to predict the *α* values from January 23 to February 14 without city lockdown. (F) Predicted *α* values and *φ* values were used to calculate the numbers of infections in China excluding Hubei province from January 24 to February 15.

Next, we predicted the increase in infections, supposing that the two measures had never been implemented. To assess the impacts of city lockdown on Wuhan, the lowest *α* value before January 23 was set as the *α* value before city lockdown, and we made the same assumption for the recovery number. Infections in Wuhan were predicted from January 24 to February 15 using simulated *α* values and *φ* values. Similar analyses were performed using data from China excluding Hubei. The infections in Wuhan city and China excluding Hubei were predicted to be 36241 and 129269, respectively, on February 15. In reality, with city lockdown, the numbers of infections in Wuhan city and China excluding Hubei were 36547 and 8533, respectively (Fig 3).

**Fig 4:**
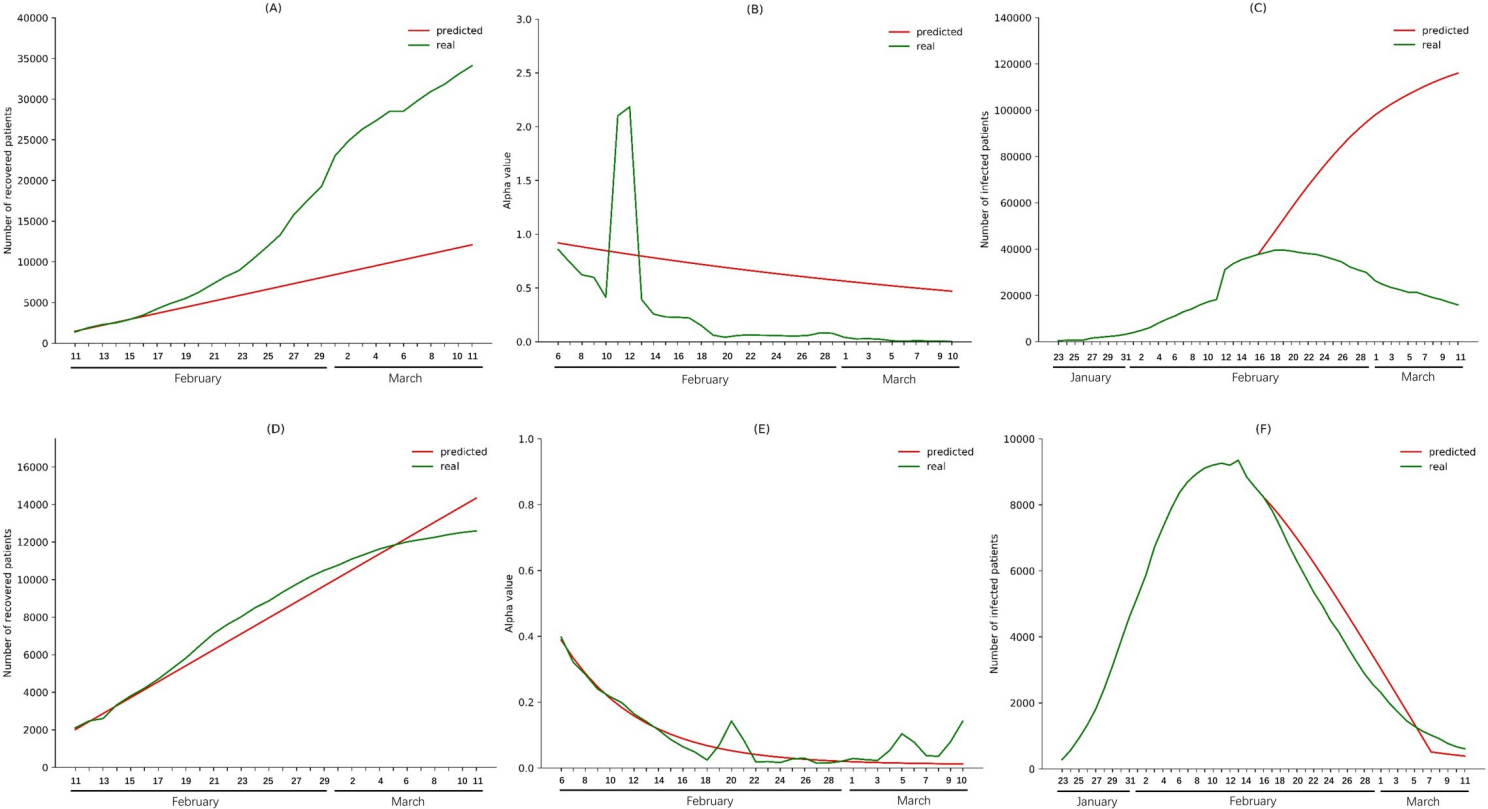
Impact of intensive community screening on Wuhan city and China excluding Hubei province. (A) The *φ* values from February 11 to February 15 (before intensive community screening) were fitted to predict the *φ* values for Wuhan city from February 16 to March 11 without intensive community screening. (B) The *α* values from February 6 to February 15 (before intensive community screening) were fitted to predict the *α* values for Wuhan city from February 16 to March 10 without intensive community screening. (C) The predicted *α* values and *φ* values were used to calculate the numbers of infections in Wuhan city from February 16 to March 11. (D) The *φ* values from February 11 to February 15 (before intensive community screening) were fitted to predict the *φ* values for China excluding Hubei Province from February 16 to March 11 without intensive community screening. (E) The *α* values for China excluding Hubei Province from February 6 to February 15 were used to predict the *α* values for China excluding Hubei Province from February 16 to March 10 without intensive community screening. (F) The predicted *α* values and *φ* values were used to calculate the numbers of infections in China excluding Hubei Province from February 16 to March 11.

Similar analyses were performed to evaluate the effectiveness of intensive community screening. For Wuhan city and China excluding Hubei, the *α* values from February 16 to March 10 were predicted using data from February 6 to February 15, and the *φ* values from February 16 to March 11 were predicted using data from February 11 to February 15. Then we used the predicted *α* values to calculate *daily*_*recover*_*num*, and infections from February 16 to March 11 were modeled using the predicted values of *α* and *daily*_*recover*_*num*. Infections in Wuhan city and China excluding Hubei were predicted to be 116003 and 388, respectively, on March 11. In reality, with intensive community screening, the numbers of infections in Wuhan city and China excluding Hubei were 15892 and 610, respectively, on March 11 (Fig 4).

### Prediction of the epidemic trend in China

**Fig 5:**
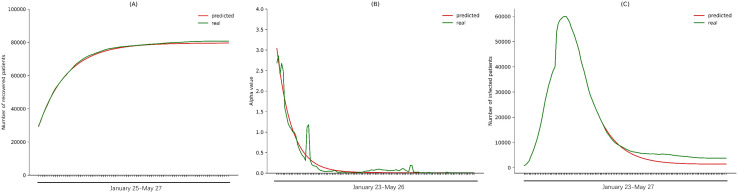
Comparison of predicted and actual numbers of epidemic patients in China. (A) The *φ* values from January 25 to March 10 were fitted to predict the *φ* values for China from March 11 to May 27. (B) The *α* values from January 23 to March 10 were fitted to predict the *α* values for China from March 11 to May 26. (C) The predicted *α* values and *φ* values were used to calculate the numbers of infections in China from March 12 to May 27.

The α values from March 11 to May 26 were obtained by fitting the α values from January 23 to March 10. We also used the higher *φ* values in the late phase of epidemic to predict *φ* values from March 11 to May 27. Taking the initial *i*(*t*), *s*(*t*), and *r*(*t*) as the values on March 10, we predicted the number of patients on a given day *i*(*t*) from March 12 to May 27. (Fig 5). We collected data on the actual number of patients in China up to May 27, and plotted the curve to compare with the predicted results for the same time period. The predicted trend showed only slight deviation from the real-world trend (Fig 5).

## Discussion

COVID-19 has become a pandemic and has led to incalculable losses around the world. At the beginning of the pandemic, little was known about the SARS-CoV-2 virus and many believed that hand hygiene was the most important way to prevent the public from being infected. The virus was reported to be able to persist on certain surfaces for hours [10], and viral RNA was even detected in patient feces [11]. As the epidemic worsened, there is evidence shown that wearing facemask by public, especially in conjunction with social distancing, is important to contain the community transmission [12]. More and more health workers and scientists suggested that people wear facemasks when entering public areas [13]. This led to a global shortage of facemasks during the early pandemic; even healthcare providers were unable to obtain sufficient N95 or surgical facemasks. This situation made social distancing crucial for halting transmission and relieving pressure on the overwhelmed healthcare system.

The Chinese government implemented the Wuhan city lockdown and nationwide intensive community screening to enforce social distancing. We were interested in studying the effectiveness of these two measures using an infectious disease model. Based on the classical SIR model in epidemiology, R0 was calculated as 2.80, which is similar to other studies [14–19]. However, this model could not be used to predict the development of an epidemic in which the government adopted drastic measures to promote isolation of infected individuals and their close contact. Therefore, we introduced here the *α* value into the SIR model, which is a scaling factor of *i*(*t*), such that *α*×*i*(*t*) indicates the number of patients who are not quarantined. The results show that this modified SIR model is highly accurate at predicting numbers of infections, based on data from Zhejiang and Guangzhou provinces.

Next the modified SIR model was used to evaluate the effectiveness of city lockdown and intensive community screening. The predicted numbers of infections without those mitigation measures were much higher than the real numbers of infections in Wuhan and China excluding Hubei, suggesting that the two mitigation measures were effective in suppressing the spread of the epidemic. A step-wise reduced *α* value may reflect effective isolation of infected individuals. Specifically, Wuhan city lockdown largely decreased the *α* value of China excluding Hubei by containing a large proportion of infected individuals in Wuhan city. Hospital beds were in extremely short supply at the beginning of the epidemic, which meant that a large proportion of infected individuals returned to the community, where they could transmit the virus to family members and neighbors. Intensive community screening, in turn, significantly decreased the *α* value locally in Wuhan city and kept it at a low level. This is probably because when screened individuals tested positive, they were notified and advised to self-quarantine at home or, if they lived with several others, to self-quarantine in newly built medical centers or quarantine facilities. At the same time, people living in close contact with the positive individual were also advised to self-quarantine. These steps appear to have been effective at reducing the *α* value.

Although our model approximated real-world data well, it deviated slightly between March and May. This may be related to imported cases, which may cause the real *α* value to exceed the predicted one. By the end of June, over 1800 imported cases had been reported in China.

Our study has limitations. First, this modified SIR model predicts the *α* values based on the current trend. If major disturbances occur in the future, such as critical virus mutation or new measures from the government, the existing model may be less accurate. Second, the two measures of city lockdown and nationwide community screening may not be suitable for every country. Some countries advocate allowing the development of herd immunity without stringent mitigations. However, this approach may be inappropriate for countries as populous as China. Many countries may not be able to achieve stringent city lockdowns or intensive community screening due to concerns over violating personal privacy and human rights. Nevertheless, it may be possible to use “softer” approaches to achieve social distancing and rapid isolation of potentially infected individuals, such as educating the public about the seriousness of COVID-19 and asking individuals with suspected symptoms to strictly self-quarantine.

Despite these limitations, our study presents a reliable modified SIR model to predict the development of the COVID-19 epidemic. Based on this model, city lockdown is effective at blocking the spread from the epidemic area into other cities, while intensive community screening may cut off infected individuals from susceptible individuals. These two measures greatly improve social distancing. Global efforts are needed to halt the pandemic. Here we shared our experiences with implementing social distancing at the national level. Other countries should tailor their social distancing strategies to their local situation.
